# Data Management: A Practical Guide for Librarians

**DOI:** 10.5195/jmla.2017.336

**Published:** 2017-10-01

**Authors:** Gerald Natal

The problem of managing the surfeit of existing research data is a growing concern. Recently published books that focus on data management in libraries employ a variety of voices and purposes. There are primers, case studies, and collections of articles written by authors in different information specialties. One recent publication, the *Medical Library Association Guide to Data Management for Librarians*, is a series of articles, with librarians working in hospitals or biomedical or health sciences institutions as the intended audience.

*Data Management: A Practical Guide for Librarians* distills much of the information found in these other books into a comprehensive plan aimed at librarians working in health sciences settings. The book is number 28 in the Practical Guides series, which are designed to provide useful information to librarians at any career level. The author is currently the director of research data management at Virginia Commonwealth University Libraries, with experience in both writing and teaching about data plans. Active in the Medical Library Association (MLA) and a member of the Academy of Health Information Professionals (AHIP), Margaret E. Henderson, AHIP, hopes the book will “inspire librarians to start providing data management services at their institutions” (p. 181).

Organized to take the reader through every step of the data management process, each chapter of the book progresses logically, from defining data through defining best practices to writing data plans and teaching about the importance of preserving data. The role of the library and librarians through all phases of data management is emphasized, and the book does a good job relating how the particular skill of the reference interview is applicable. Librarians who are inexperienced in teaching will find that the chapter on “Teaching Data” provides useful advice for teaching any subject. References and a key-point summary support each chapter, along with a glossary of terms for anyone who may be new to the concept of data management. There are plenty of examples, figures, and bulleted lists to illustrate and summarize points. All these elements combine to make for an informative and enjoyable read.

*Data Management: A Practical Guide for Librarians* should be in the hands of any librarian who seeks to find solid background information on data management. Those who seek to establish a plan will find a detailed outline of goals, objectives, aims, theories, policies, technical considerations, and best practices. That the book was written from the perspective of a health information professional and experienced data manager may provide extra appeal for librarians in the health sciences.

